# Challenges in the diagnosis of dementia: insights from the United Kingdom-Brazil Dementia Workshop

**DOI:** 10.1590/1980-57642020dn14-030001

**Published:** 2020

**Authors:** Victor Calil, Emma Elliott, Wyllians Vendramini Borelli, Breno José Alencar Pires Barbosa, Jessyka Bram, Felipe de Oliveira Silva, Leonardo Galvão Machado Cardoso, Luciano Inácio Mariano, Natalia Dias, Michael Hornberger, Paulo Caramelli

**Affiliations:** 1 Instituto D’Or de Ensino e Pesquisa - Rio de Janeiro, RJ, Brazil.; 2 Universidade Federal do Rio de Janeiro - Rio de Janeiro, RJ, Brazil.; 3 Institute of Cardiovascular and Medical Sciences, University of Glasgow - Glasgow, United Kingdom.; 4 Faculdade de Medicina, Pontifícia Universidade Católica do Rio Grande do Sul - Porto Alegre, RS, Brazil.; 5 Instituto do Cérebro do Rio Grande do Sul - Porto Alegre, RS, Brazil.; 6 Instituto de Medicina Integral Prof. Fernando Figueira - Recife, PE, Brazil.; 7 Departmento de Neurologia, Hospital das Clínicas, Universidade de São Paulo - São Paulo, SP, Brazil.; 8 Laboratório de Neurociências, Departamento e Instituto de Psiquiatria, Universidade de São Paulo - São Paulo, SP, Brazil.; 9 Instituto de Psiquiatria, Universidade Federal do Rio de Janeiro - Rio de Janeiro, RJ, Brazil.; 10 Faculdade de Medicina, Universidade Federal da Bahia - Salvador, BA, Brazil.; 11 Programa de Pós-Graduação em Neurociências, Universidade Federal de Minas Gerais - Belo Horizonte, MG, Brazil.; 12 Laboratório Interdisciplinar de Investigação Médica, Faculdade de Medicina, Universidade Federal de Minas Gerais - Belo Horizonte, MG, Brazil.; 13 Norwich Medical School, University of East Anglia - Norwich, United Kingdom.; 14 Departamento de Clínica Médica, Faculdade de Medicina, Universidade Federal de Minas Gerais - Belo Horizonte, MG, Brazil.

**Keywords:** dementia, diagnosis, cognitive impairment, biomarkers, neurobehavioral manifestations, demência, diagnóstico, disfunção cognitiva, biomarcadores, manifestações neurocomportamentais

## Abstract

In July 2019, a group of multidisciplinary dementia researchers from Brazil and the United Kingdom (UK) met in the city of Belo Horizonte, Minas Gerais, Brazil, to discuss and propose solutions to current challenges faced in the diagnosis, public perception and care of dementia. Here we summarize the outcomes from the workshop addressing challenges in diagnosis. Brazil faces a major problem in dementia underdiagnosis, particularly involving the population in an adverse socioeconomic context. There is poor availability of resources and specialists, and the knowledge of general practitioners and other healthcare professionals is far from satisfactory. Low education level is a further obstacle in diagnosing dementia, as the most commonly used screening tests are not designed to evaluate this population. Patients and their families must overcome the stigma of a diagnosis of dementia, which is still prevalent in Brazil and increases the burden of this condition. Whilst the UK has greater resources, dedicated memory services and a National Dementia Strategy plan, the National Health Service (NHS) has limited funding. Therefore, some challenges regarding diagnosis are common across both countries. The authors suggest possible solutions to confront these, with the goal of improving assessment and recognition of dementia and reducing misdiagnosis.

## RESUMO.

Em julho de 2019, um grupo multidisciplinar de pesquisadores em demência do Brasil e do Reino Unido se reuniu em Belo Horizonte para discutir e propor soluções para os desafios no diagnóstico, percepção pública e tratamento dessa condição. Neste artigo, sintetizamos as conclusões do *workshop* sobre os desafios no diagnóstico de demência. O Brasil enfrenta um grande problema no subdiagnóstico de demência, principalmente entre a população em condições socioeconômicas adversas. Há pouca disponibilidade de recursos e de especialistas e o conhecimento de médicos generalistas e de outros profissionais de saúde é pouco abrangente. Baixa escolaridade é também um obstáculo no diagnóstico de demência, uma vez que os testes de rastreio mais utilizados na prática clínica não são estruturados para avaliar a população com esse perfil. Os pacientes com demência e suas famílias ainda têm que superar o estigma do diagnóstico, que é ainda muito prevalente no Brasil e colabora para a piora da qualidade de vida. O Reino Unido, por outro lado, dispõe de mais recursos financeiros e de pessoal, possui serviços dedicados à avaliação de problemas de memória e um plano estratégico nacional para demência. Contudo, o *National Health Service* (NHS) tem verbas limitadas, o que faz com que alguns dos desafios no diagnóstico de demência sejam comuns aos dois países. Os autores sugerem possíveis soluções para enfrentá-los, com o objetivo de melhorar a avaliação e o reconhecimento da demência e reduzir os erros de diagnóstico.

## INTRODUCTION

The upcoming decades will see a rise in the prevalence of dementia, especially in developing countries. Therefore, healthcare systems must prepare to provide timely and accurate diagnosis and have the resources in place for efficient care. There are many barriers to reach this goal, and they vary in places with a lower socioeconomic status. An important step to address this is to share experience and best practice with professionals from other states and countries, establish collaborations and work together towards feasible, creative solutions.

The United Kingdom-Brazil Dementia Workshop took place in the city of Belo Horizonte, State of Minas Gerais, Brazil, on July 25-27, 2019, with participation of researchers from both countries, and aimed to facilitate collaborative and creative solutions to address challenges in public perception of dementia, diagnosis and care management. This article summarizes the discussions from the workshop regarding challenges in the diagnosis of dementia, both in Brazil and in the United Kingdom (UK). We also provide some suggestions to overcome these barriers, with the goal of improving the assessment and recognition of dementia and reducing misdiagnosis.

With the fifth largest population in the world, Brazil faces extreme socioeconomic inequality. Rich, cosmopolitan metropoles deal with a very different reality than small, remote or indigenous communities regarding access to healthcare (both primary and specialist, tertiary care) and availability of diagnostic resources. Even in the largest cities, there are important inequalities, for example, when we compare peripheral areas to rich neighbourhoods. Though there are data on prevalence of dementia in the richer, southernmost regions of Brazil,^1^ ^,^ ^2^ ^,^ ^3^ there is no information, i.e. population-based studies, for other regions. Due to this heterogeneity, approximately 77% of people with dementia are left undiagnosed in Brazil.^4^ As a comparison, since 2015 two-thirds of individuals with dementia in the UK have had a formal diagnosis.^5^ At the other end of the spectrum, misdiagnosis is also a problem, as many individuals in Brazil receive an incorrect diagnosis of a cognitive disorder. There are guidelines for diagnosis, both in Brazil and in the UK,^6^ ^,^ ^7^ yet they have not been converted into practice in the Brazilian public healthcare system.

Around 850,000 people are estimated to be living with dementia within the UK.^8^ The UK Government and the National Health Service (NHS) have made dementia a priority and, since the first National Dementia Strategy was published in 2009, improvements have been demonstrated in both dementia services and funding into dementia research. An example thereof is the increase of diagnostic rates since the launch of a national policy.^9^ Memory clinics are an essential part of this strategy, serving as the cornerstone of diagnosis for people with suspected dementia and as gatekeepers to treatment and post-diagnostic support. Access is usually restricted to referral. They have been around since the early 1980s and have spread ever since to a current number of 222 in England.^10^ Memory clinics are also extremely important because the staff there have the most expertise; they are delivered by specialist multidisciplinary teams and are usually led by a Psychiatrist or Neurologist specialized in old age. Nevertheless, the NHS is substantially stretched and psychiatric services are often hit the worst. The UK is currently facing a shortage of psychiatrists and the effect this will have on services is worrisome.

Besides that, waiting lists are often long, and for those who live in remote areas, access may not be feasible. It can, therefore, take a long time to reach a diagnosis of dementia, which is also an issue in Brazil. This should not always be viewed negatively, since a range of investigations needs to be carried out and differential diagnoses must be considered. In the UK, diagnosis and support of vascular dementia need particular improvement, since many patients are turned away from memory clinics if the cognitive decline is deemed vascular in aetiology, for example if they have a history of stroke. This is mainly due to the uncertainty regarding trajectory of vascular cognitive impairment.

### Challenges in the diagnosis of cognitive impairment and dementia in primary care

The General Practitioner (GP) plays a crucial role in the pathway to receiving a diagnosis, therefore it is sensible to start with the challenges faced in the primary care setting. In the UK, GPs are encouraged to identify dementia cases, but they are not considered responsible for making a formal diagnosis. Suspected cases are referred onto memory clinics, and the clinicians there make the diagnosis. Reversible causes of cognitive decline, such as infection, metabolic disorders or depression, should be investigated by the GP and ruled out before specialist referral is made. An incorrect label of dementia can cause both physical and psychological harm, so it is crucial that healthcare professionals are aware of common dementia mimics, including functional cognitive disorders, which are frequently seen by memory clinics. One study found that more than 50% of memory clinic attendees had a functional cognitive disorder rather than dementia.^11^

Diagnosis of rarer forms of dementia, for example non-amnestic presentations of Alzheimer’s disease (AD), frontotemporal dementia, and primary progressive aphasia (PPA), can be especially delayed, since there are fewer healthcare professionals with experience in recognizing these conditions, and fewer specialist centres with expertise. Moreover, in the Brazilian public health care system the access to neuroimaging tools, particularly magnetic resonance imaging (MRI), is still limited to larger cities. Also, the disconnection between different levels of care needs to be improved. Since the process of receiving a diagnosis can be timely and involves numerous stages, it is important that patients and families are kept informed of the timelines, what investigations are taking place, and why they are happening.

#### Challenges in screening

The problem with diagnosing dementia in Brazilian primary care starts at the very first step of memory complaints evaluation: cognitive screening. Medical appointments tend to have a very short duration in this setting, sometimes less than ten minutes, and must comprise all kinds of health issues, some of which are much more compelling than memory complaints. Even when the GP assesses cognition, the limited time makes detailed cognitive tests not doable, which makes shorter, simpler tests more suitable. However, health professionals in Brazil are usually not aware of different instruments for dementia screening and are not trained to perform them.

Appointment length is not so dissimilar within primary care in the UK, with duration also affecting feasibility of cognitive screening. A GP will usually have to see the patient over multiple appointments to gather collateral history, carry out cognitive screening and report back results of cognitive and blood tests. Cognitive test choice is ultimately down to the individual GP’s preference. A recent survey found the three tests most commonly used by British GPs are the Mini-Mental State Examination (MMSE), GP Assessment of Cognition (GPCOG), and the 6-Item Cognitive Impairment Test (6CIT).^12^

In Brazil, the MMSE^13^ is the most commonly used tool, as suggested by the Brazilian Ministry of Health Protocol for Alzheimer's Evaluation.^14^ The use of MMSE, however, is not fully standardized, not only because of the lack of training, but also due to the frequent use of cultural adaptations. This happens with the aim to overcome differences between international samples and the Brazilian population (e.g. replacing “season” with “semester” or “time”) and to reduce floor effects related to educational bias (e.g. serial additions of “5” instead of subtractions of “7” and spelling “Maria” backwards instead of “world”).^15^ Another problem with the MMSE is the big influence of education on the performance of patients and the different cut-points suggested in the literature.^15^ Despite these limitations, the MMSE is useful for initial cognitive screening in Brazil.

Other common challenges which are not country-specific include testing individuals with sensory loss who forget to bring along their glasses/hearing aid, so it is crucial that healthcare professionals remind and stress the importance of bringing them to the appointment. Environmental factors also affect assessment. The importance of having a relaxed, quiet atmosphere is emphasized for an accurate, reliable cognitive assessment. A rushed GP appointment may therefore affect performance.

The best way to improve the screening of dementia in primary care is to establish protocols using simple and short tests. Tests should be standardized, regarding not only the method of evaluation, but also normative values. Ideally, the screening instruments should not be heavily influenced by educational level. After that, health professionals should be trained to choose and use the tests properly.

There are a number of tests available which can be administered in under ten minutes, for example some shortened versions of the Montreal Cognitive Assessment (MoCA) have been found to have high sensitivity, similar to that of the full scale.^16^ Test choice should be informed by psychometric properties and the available resources in the service one is referring onto. For example, highly sensitive tests are generally preferred (so that dementia cases are not missed); however, these come at a cost of lower specificity (more false positive cases). If this does not suit the service, then a test with a higher specificity may be preferred. Evidence-based guidance exists for test choice within a primary care setting.^17^

Informant-based assessments, such as the Informant Questionnaire on Cognitive Decline in the Elderly (IQCODE),^18^ may also provide additional useful information, but are often overlooked both clinically and in research.^19^ The questionnaire can be filled out by someone close to the patient to evaluate whether there has been any change in cognition over time. The ideal would be to have this information in addition to the patient’s cognitive evaluation.

#### Who should screen and diagnose?

In the UK, cognitive screening is carried out by a range of healthcare professionals: physicians, nurses, occupational therapists, and psychologists. Although screening tests are designed for non-specialists, training should not be taken for granted. Ambiguous or incorrect scoring has been found both in clinical practice and research.^20^ Formal diagnosis is usually made by specialists (geriatricians, neurologists or psychiatrists).

In Brazilian primary care, diagnosis of dementia rarely comes from a multi-disciplinary assessment, given that physicians are usually the only professionals trained to perform cognitive screening. Training other healthcare professionals to use screening instruments correctly is a feasible solution. Community health agents could also be a useful alternative, as they benefit from increased contact with the local population. Scales and interviews with proxies, such as the IQCODE,^18^ could be administered by these agents. Moreover, simple and basic guidelines or flowcharts could be used to enable these professionals to raise suspicion of pathological aging and refer patients with memory complaints to specialists.

Another reason for underdiagnosis of dementia in Brazilian primary care is the lack of confidence of GPs to diagnose these conditions. Most physicians are not aware of the guidelines and protocols for evaluation and management of patients with memory complaints, such as the Brazilian Ministry of Health protocol.^14^ On the other hand, there is little availability of specialists (neurologists, psychiatrists, geriatricians) in the public health system, particularly in smaller cities or in rural areas. Communication between GPs and specialist teams also needs to be improved. For some cases it would be beneficial if GPs received more training and were able to take a greater role in providing a diagnosis of dementia. For patients in remote areas unable to attend to see their GP or attend specialist appointments, telemedicine consultations should be considered. There are cognitive screening tests specifically developed to be delivered over the telephone, for example the Telephone Interview for Cognitive Status (TICS).^21^

### Challenges in diagnosis of cognitive impairment and dementia in populations with low education and immigrants

Although low education level is not considered a common issue in the UK, the increase of immigration in the past few years has raised concern about the development and diagnosis of dementia in adverse socioeconomic situations. Healthcare professionals are usually not capable of interpreting cultural norms or preferences and may not acknowledge differences both between and within immigrant groups, thus jeopardizing diagnosis and management of these patients.^22^ As a consequence, individuals from minority ethnic groups tend to be diagnosed later and are less likely to receive medications for dementia.^23^ ^,^ ^24^

Low education and poverty are among the greatest challenges in the Brazilian population. Patients in these categories not only have a higher risk of developing dementia, but also have poorer access to health services and diagnostic tests. They also face geographical obstacles, as proper healthcare rarely arrives in remote places, such as the indigenous communities in the Amazon or the poorer towns in the semi-arid region of the Brazilian Northeast. Similar issues are still present within the UK in areas with a lower socioeconomic status, but on a much smaller scale than that of Brazil.

Most of the studies regarding dementia in low education patients were performed in developed countries, where individuals with less than five years of formal study are rare. Hence, there is little evidence on how to properly diagnose these patients. The most commonly used screening tools, such as the MMSE and the MoCA, are influenced by educational level and may yield a high number of incorrect diagnoses. There are some tools devised to solve this problem, such as the Rowland Universal Dementia Assessment Scale (RUDAS),^25^ which has been shown to have high specificity across cultures in immigrant populations and to be less affected by education and language than the MMSE.^26^ Some of the classic screening tools also have versions to test individuals with low education, such as the MoCA-Basic test.^27^ Another option is the Brief Cognitive Battery (BCB),^28^ which is also not significantly influenced by education and has been used by different groups in Brazil and in other Latin-American countries.

The first step to diagnose dementia among immigrants and people with low education is to recognize the important cultural peculiarities that may serve as a barrier in these populations. As an important example, some people in Brazil and in immigrant communities in the UK may understand symptoms of dementia as a religious (sometimes even as a punishment) or a pejorative issue, thus delaying help-seeking and treatment.^29^ Community agents may play a pivotal role in this context, as they generally belong to the same population as the patients they assess and tend to have a better understanding of their cultural particularities than a physician.

Education and training of health professionals is a priority, particularly to recognize dementia in individuals with low cognitive demand. The most common screening methods have important limitations, so that it is very important that providers are aware of the different cut-points and of instruments less affected by education. It is also essential to raise dementia awareness amongst the general population, so people know when they should seek help. This can be done through soap operas, social networks or national campaigns, ideally with simple, straightforward messages.

### Clinical vs. biological approach of dementia

In recent years, the emergence of cerebrospinal fluid (CSF) and neuroimaging biomarkers has shifted the definition of dementia from a clinical to a biological perspective.^30^ This change of framework has been suggested only for research purposes and not yet for clinical use, as there is currently no specific treatment affecting the molecular basis of AD. Nevertheless, the availability of biomarkers is already changing the way physicians treat patients with suspected AD. More concretely, the IDEAS Study^31^ showed that the use of amyloid imaging with positron emission tomography (PET) was associated with changes in the clinical management of patients with mild cognitive impairment (MCI) and dementia, even though there is still no evidence that current Alzheimer drugs are more efficient in patients with positive biomarkers.

It is still too early to presume the impact of this change of paradigm in public health. The biological definition of AD will substantially increase diagnosis, including asymptomatic individuals with pre-clinical disease.^32^ This might be an opportunity to intervene in early phases of dementia when targeted disease-modifying treatment for AD are available. Until this happens, however, the high prices of AD biomarkers make their cost-effectiveness debatable, particularly in low- and middle-income countries. More importantly, the evidence supporting biomarker-based diagnosis is not yet definitive.^33^ Labelling healthy individuals with AD pathology as having the disease even though they may never develop cognitive impairment^33^ would significantly raise healthcare expenses, expose individuals to adverse effects of treatment and have important legal implications.^34^

The Brazilian protocol for diagnosis of dementia^14^ suggests that the basic evaluation of patients with memory complaints should include neuroimaging - either computed tomography (CT) scan or MRI - and blood tests for exclusion of reversible dementias. Nevertheless, it may take several months for patients in public health to get a simple brain CT scan and even longer to get an MRI. In Brazil, AD biomarkers analysis is currently restricted to research centres in universities or private clinics. Most of the patients who would benefit from such testing (i.e. early onset, atypical presentations), however, do not have access to these centres or cannot afford their high cost. In the UK, CT scans are organized by the memory clinic. Beyond this, further imaging is generally only recommended where symptoms are rapidly progressing or where diagnosing a dementia subtype is considered beneficial and knowledge of this would change management. Therefore, they are not routinely undertaken.

Interpretation of AD biomarkers is still a problem, since there may be significant differences between methods and techniques. Moreover, there is a concern that misinterpretation of these tests might increase the prevalence of false diagnosis, particularly if they are taken out of clinical context. We suggest that such tests should only be asked and interpreted by specialists.

Another relatively new tool in dementia diagnosis are genetic tests. Genetic tests might be useful for the diagnosis of hereditary forms of dementia or to recognize high-risk individuals. This kind of evaluation is still restricted to some university centres and major cities and are not available in the public health system. In order to avoid misdiagnosis, we also believe that only specialists should require such testing.

### How does the diagnosis affect the patient and the family? Diagnostic disclosure and stigma issues

In a systematic review comprising studies of several countries, more than 90% of individuals without cognitive impairment are in favour of diagnostic disclosure, as opposed to 85% of patients with memory issues.^35^ In the UK, once someone has received investigations and specialist input, they would rarely request a diagnosis to be withheld. In Brazil, however, the view is traditionally different. Family members and caregivers often ask physicians to not reveal the diagnosis to the patients: only 58% of relatives of patients with dementia in Brazil are in favour of diagnostic disclosure, though 90% of them would like to be aware of the diagnosis.^36^ Moreover, only 45% of Brazilian medical specialists who commonly diagnose and treat individuals with dementia reported that they regularly disclose the diagnosis to patients.^37^ The Brazilian guideline suggests that the approach on this issue should be individualized and should take into account cultural, regional and individual factors.^6^

Part of this interesting phenomenon can be explained by the stigma of dementia. In Brazil, cognitive decline is often viewed as a normal part of aging. People avoid saying “dementia” or “Alzheimer’s” aloud due to its negative connotations. As pointed before, some view it as a pejorative concept. Most individuals also have little or no knowledge about the progression of dementia, what to expect and what can be done to mitigate the problems to come. Stigma seems to be worse among high-income families, that would sometimes feel ashamed and hide the condition from other people, as opposed to lower-income families, that frequently depend on a network of relatives, friends, and neighbours to solve their problems, e.g. to take care of the patient.

Stigma can only be tackled through education. It is essential to raise awareness of dementia in the general population, in order to reduce false preconceptions. The media and public figures are invaluable in achieving this goal. Health professionals need to be trained as well to adapt their approach to patients and families according to individual cultural idiosyncrasies that may lead to inaccurate perceptions of the disease. Most importantly, the provider needs to thoroughly explain the degenerative nature of dementia and what to expect in the years to come.

### Recommendations

There are several challenges to ensure timely and accurate diagnosis to the majority of patients with dementia (Table 1), yet some of them may be overcome through training (Box 1). It is vital to train healthcare professionals to recognize signs of pathological aging, to know which screening methods to use and how to apply them. They also need to know how to approach patients with different cultural backgrounds and how to reduce stigma by properly instructing patients and their families at the time of diagnosis.

**Table 1.** Challenges of dementia diagnosis in the United Kingdom (UK) and Brazil.

UK	Brazil
Long waiting lists to attend memory clinics	Memory clinics restricted to universities
Shortage of staffing, especially psychiatrists and psychologists	No National dementia strategy plan
Insufficient funding	Poor access to neuroimaging
Short appointment length in primary care	Short appointment length in primary care
Insufficient training for non-specialists	Non-specialists rarely perform cognitive screening
Minority ethnic groups and immigrants tend to be diagnosed later	Limitations in the diagnosis of dementia in low education patients
Stigma of dementia patients and their families	Stigma of dementia patients and their families

**Box 1.** Recommendations to address challenges in diagnosis.

Screening protocols with simple and short tests
Training healthcare professionals to accurately perform cognitive screening
Training GPs to diagnose patients with dementia
Enable community health agents to recognize signs of cognitive decline
Telemedicine consultations could improve access to specialists
Training healthcare professionals to recognize cultural peculiarities of immigrant communities
Use of screening methods less affected by education
Raise dementia awareness amongst the general population, through soap operas, social networks or national campaigns
AD biomarkers and genetic tests should only be interpreted by specialists
Reduce stigma by improving communication between provider and patients/families

GP: general practitioner; AD: Alzheimer’s disease.

Community agents should be viewed as a key part of the efforts to improve dementia diagnosis and care. These professionals are usually from the same neighbourhoods as the patients to whom they attend and share similar cultural characteristics. If properly trained, they can help the dementia screening process by recognizing memory complaints and may serve as a link to ease the communication between patients with low education or from specific backgrounds (e.g. immigrants) and health care professionals.

There are undoubtedly many structural flaws in Brazilian health care, given the financial situation of the country and its vast dimensions. However, we have good examples to follow, such as the *Mais Vida* Program, in the state of Minas Gerais, which aims to ease access to health care for the elderly population by creating smaller centres with specialized professionals.^38^ Another interesting model happens in the UK, where charities and private organizations play an important role in the diagnosis and in the follow-up of individuals with dementia.

Most of the challenges in dementia diagnosis faced in Brazil may be reduced by a long-awaited national plan, focused on the improvement of diagnosis and raising awareness of dementia. This is further justified since challenges faced by the UK have improved after implementing such a plan. It could also set a framework for quality outcomes, including acceptable waiting times for diagnostic tests or for evaluation by a specialist.
